# MO-NanoDatabase: A metal-oxide nanostructured compound dataset composed of a huge number of metal-oxide nanocompounds, their global properties and 3D-structure

**DOI:** 10.1016/j.dib.2025.111476

**Published:** 2025-03-22

**Authors:** Francesc Serratosa, Natàlia Segura-Alabart

**Affiliations:** Universitat Rovira i Virgili, Tarragona, Catalonia, Spain

**Keywords:** XYZ format, Metal-oxide nanocompound, 3D structure, Toxicity prediction, Chemical activity

## Abstract

This paper presents the first important recompilation of metal-oxide nanocompounds, which is composed of three main parts: the first one includes several global properties of the nanocompounds whereas the second one includes their 3D structure, represented by the well-known XYZ format. Finally, the third part includes the structural nano QSAR named NanoFingerprint of these 3D structures. Modelling size-realistic metal-oxide nanomaterials to analyse some of their properties, such as chemical activity, solubility, or electronic structure, is a current challenge in computational and theoretical chemistry. Several nano QSAR models have been published based on global properties of these compounds, but few QSAR models also leverage their 3D structure. A general database of nanocompounds is crucial for the validation of current and future models.

The global properties have been extracted from datasets published as the supporting material of papers that present new models for property prediction of metal-oxide nanocompounds [2–7]. The data has been curated, imposed the same units, formatted and given the same name per property since we realised the low generalisation on units, formats and nomenclature. Note the input parameters of the QSAR models and also the properties to be predicted have been put together as global properties in our database. Moreover, the 3D crystallographic structure has been computed through simulation computer applications of all the compounds since these structures could not be found in most of the cases.

Since it is the first time that all this knowledge is compiled in a unique database, the purpose of MO-NanoDatabase is to be a reference database for prediction (chemical activity, solubility or electronic structure) of metal-oxide nanocompounds for current and future nano QSART models. Although many nanocompounds have been included, new versions of the database are not discarded if they bring substantial quantity of new nanocompounds presented in future papers.

Specifications Table

**Table:** 

Subject	Nanotechnology.
Specific subject area	Nano QSAR modelling for metal-oxide property prediction based on global properties and 3d crystallographic structure. The main properties of the nanostructured compounds depend on the original source as follows: [2]: Energy (Kcal eqv-1), Zeta potential in water (mV), Isoelectric point [3]: Mulliken'selectronegativity (eV), Enthalpy of formation of a gaseous cation (kcal/mol) [4]: Size in water (nm), Zeta potential in water (mV), Zeta potential (KCI) (mV) Size in water (nm), Size in PBS (nm), Concentration, Zeta Potential, LDH response, [5]: hydrosize (nm), surfcharge (eV), surfarea (nm2), Hsf, Ec, Ev, MeO, Cellline, Cancer Cell (Yes/No), Exposition time, Dosage, Enthalpy, Ratio, e, esum, esumbyo, MW, Viability, Toxic (Yes/No) [6] Energy (Kcal eqv-1), Zeta potential in water (mV), Isoelectric point
Type of data	One Table of global properties in Excel format. A list of XYZ files. A list of NanoFingerprint files.
Data collection	**Global properties**: A process of curation and standardisation was applied to the global properties extracted from the supporting material of the papers mentioned in Reference section. Samples in these papers that properties were missing or incongruent were discarded. Moreover, not all papers presented all properties. **3D structure (XYZ file):** Generated by the public crystallographic tool in: https://nanocrystal.vi-seem.eu and reported in [7]. **NanoFingerprints:** Generated by https://atena.urv.cat/model/
Data source location	Universitat Rovira I Virgili, Tarragona, Catalonia, Spain
Data accessibility	Repository name: MO-NanoDatabase 10.5281/zenodo.13986574 Direct URL to data: https://github.com/FrancescSerratosa/MO-NanoDatabase
Related research article	Francesc Serratosa, “GraphFingerprint: Graph Embedding of graphs with almost constant sub-structures”, Pattern Analysis and Applications, 2024. 10.1007/s10044–024–01366-w

## 1 Value of the Data

• Although metal-oxide nanocompounds have multiple applications (for instance, agriculture, thermal isolation, biotechnology), there is a problem of lack of truthful data and non-standardisation of this data. This database will be useful to verify the current prediction models and to define new ones. The aim is to make this database to be the core of metal-oxide nanocompound predictive models benchmarking.
• Researches in public Universities, technological centres and private companies will have a public and standardized database to test their predictive models of metal-oxide nanocompounds.
• Chemical labs could predict the chemical activity of their nanocompounds by generating new models or comparing the new nanocompounds to the ones in the database with their properties.

## 2 Background

The aim of European projects NanoInformatix (H2020- NMBP-14–2018–814,426) and SbD4Nano (H2020-NMBP-TO-IND-2019–862,195) was to analyse, organise, cure, generate and make public as much data related to chemical nanocompounds and their chemical activity levels as possible. In this case, data means global properties of nanocompounds and also predictive models on these nanocompounds. On this framework, the NanoQSART called **NanoFingerprint** [1], was described, which specifically models a metal-oxide nanocompounds. For the first time, the 3D crystalline structure was taken into consideration into a nanoQSART. In these projects, as much global properties as possible of nanostructured compounds were collected and also, their 3D structure. The current paper shows the data that was generated and makes it public. In this way, users can predict their metal-oxide nanostructured compound chemical activity based on several models [[2], [3], [4], [5], [6], [7]] and define new models given the collected data.

**Fig. 1:**
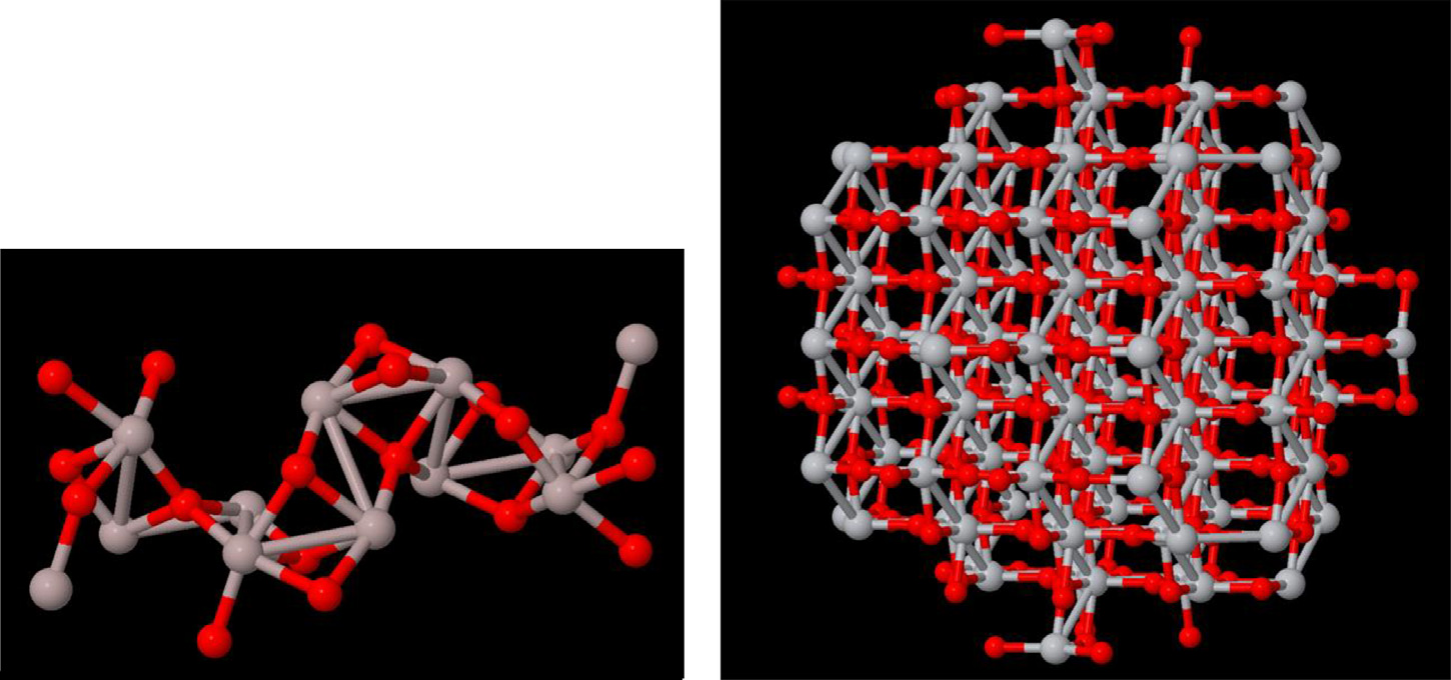
Left: Al_2_O_3_ of size 13 angstroms. Right: TiO_2_ of size 20 angstroms.

**Fig. 2:**
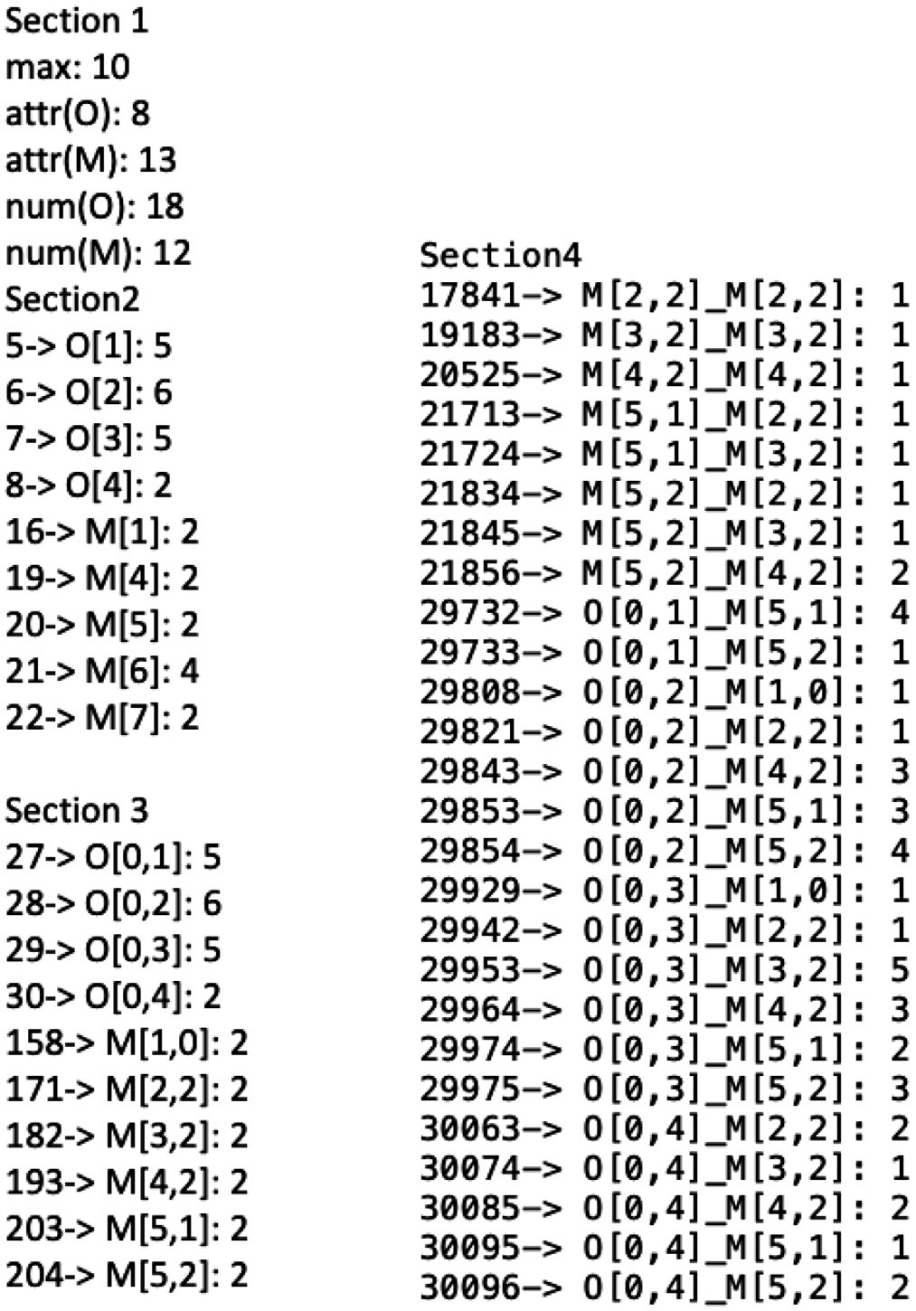
NanoFingerprint of Al_2_O_3_ of size 13 Angstroms, which is described in four sections. The left column shows the first three sections, and the right column shows the fourth section. Section 1 is composed of five numbers, viz. the max number of bonds per atom, the mass of oxygen, the mass of the metal, the number of appearances of oxygen and the number of appearances of the metal. In sections 2 to 4, the first number is the position in the embedding vector, then, after the symbol →, there is the local structure located in this position and finally, the last value is the number of appearances of this local structure. Note the positions in the embedding vector with a zero do not appear in this representation.

Fig. 1 shows two nanocompounds in the database. The left one is an Al_2_O_3_ of size only 13 Angstroms and the right one is a TiO_2_ of size 20 Angstroms. Oxygen atoms in red and aluminium and titanium atoms in grey. Moreover, Fig. 2 shows the NanoFingerprint that represents Al_2_O_3_ of size 13 Angstroms. NanoFingerprint are described in four sections (details in [1]). Through these sections, this embedding counts the number of appearances of some local structures. Table 1 summarises these structures.

**Table 1:** Local structures considered by the nanofingerprint embedding.

Position	Global features
O(x)	It represents a node type O that have x edges
M(x)	It represents any node of type M that have x edges
O(x,y)	A local structure composed of a central node of type O connected to x nodes of type O and y nodes of type M. Note these x O and y M could be connected to other nodes
M(x,y)	Similarly, a local structure composed of a central node of type M connected to x nodes of type O and y nodes of type M
O(x,y) - O(x',y')	A structure that is composed of two of the previous ones. It is composed of an O(x,y) and an O(x',y') whose central O are connected by an edge
M(x,y) - M(x',y')	In a similar way, it is composed of an M(x,y) and an M(x',y') whose central M are connected by an edge
O(x,y) - M(x',y')	Finally, a similar structure but that connects an O(x,y) and an M(x',y') whose central nodes are connected

**Fig. 3:**
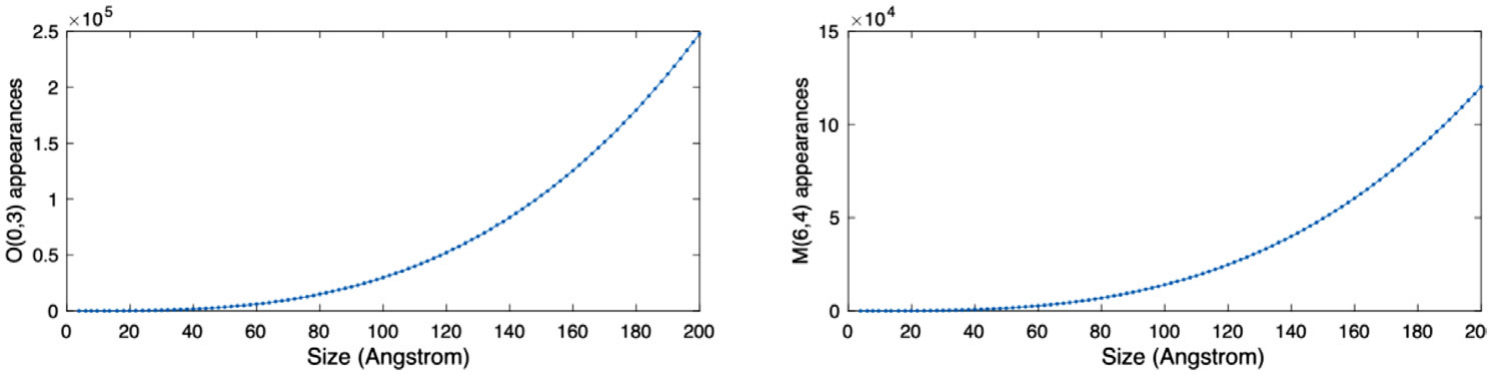
Number of appearances of O(0,3) and M(6,4) in the nanocompound TiO_2_ when its size ranges from 2 Angstrom to 200 Angstrom.

Fig. 3. Shows, as an example, the number of appearances of four different local structures, viz. O(3), M(1,0), O(0,3) and M(6,4) in the nanocompound TiO_2_ when its size ranges from 2 Angstrom to 200 Angstrom. This information is easily extracted from the NanoFingerprint Nano QSART embedding and can be used by the models to detect chemical activity levels.

## 3 Data Description

The data is composed of 564 metal-oxide nanostructured compounds, which have sizes from 7.5 Angstroms to 125 Angstroms. Metals are Al, Cu, Fe, Ti and Zn. They are described in the Excel document MO-NanoDatabase.xlsx and three folders: XYZ, NanoFingerprint and NanoFingerprint_reduced. All of it composes the repository. The information that they contain is explained below.

**MO-NanoDatabase.xlsx** is an Excel document that describes in the first sheet, which nanocompounds have been collected, which properties have been collected and from which paper have been extracted. In the rest of the sheets, there is the data extracted from each paper per sheet. In these sheets, there is exactly all collected data per all nanocompounds.

**Folder XYZ** contains the 3D structure of all the nanocompounds, which its data is in MO-NanoDatabase.xlsx.

Folders **NanoFingerprint** and **NanoFingerprint_reduced** contain the Fingerprint information of all the nanocompounds, which their data is in **MO-NanoDatabase.xlsx**. The difference between them is that folder **NanoFingerprint** exactly contains the NanoFingerprint in the original format. Contrarily, folder **NanoFingerprint_reduced** contains the NanoFingerprints but the empty cells of the NanoFingerprint vector have been removed.

## 4 Experimental Design, Materials and Methods

The recompilation and generation of the data was carried out in three steps: Collecting the samples of nanostructured compounds, generating the 3D structure and generating the NanoFingerprint.

### 4.1 Collecting the Samples

An exploration in Web of Science (WoS) was done searching for papers in topic “metal-oxide toxicity prediction” in November 2024. WoS returned 179 results. From these papers, 47 of them were discarded that the document type was classified as “Other” by WoS. Thus, leaving only 132 papers that their type of document was “Article” (160), “Review Article” (29), “Dissertation Thesis” (11) or “Book” (6).

From these 132 papers, a thorough exploration was carried out to discover which nanostructured compounds experimented with and which properties they had. Only the papers that presented the compound and their properties in the original paper or support material were finally selected.

Only five papers appeared with these conditions:

1. Search topic in WoS: topic “metal-oxide toxicity
2. No “Other” type in WoS
3. Table composed of samples. Each sample is a specific nanostructured compound with its properties.

From these papers we generated the list in **MO-NanoDatabase.xlsx** composed of 564 metal-oxide nanostructured compounds.

### 4.2 Generating the 3D Structure

Some of these 564 samples share the same 3D structure. This is because they are the same nanostructured compound but in a different external condition. For instance, temperature or pressure, which could return different toxicity or other properties. Note different sizes of the same nanostructured compound generates different 3D structures. In our case, the total number of 3D structures was 60.

The 3D structure was generated by the public crystallographic tool in: https://nanocrystal.vi-seem.eu reported in [7]. These structures are in the repository in the **XYZ** directory.

### 4.3 Generating the Nanofingerprint

Finally, the embedding of the 60 3D structures into a NanoFingerprint was performed by the tool in: https://atena.urv.cat/model/ reported in [1]. This tool only needs the 3D structure to be introduced as a .xyz file and returns a .txt file with the embedding. Nevertheless, two options can be considered, the first one is to generate the embedding (these embeddings are in the repository in the **NanoFingerprint** directory) and the second one is to generate the reduced embedding (these embeddings are in the repository in the **NanoFingerprint_reduced** directory). The second case is a compressed embedding in which the empty values are not explicitly incorporated.

## Limitations

Not all the papers describe the same properties per all nanocompounds. Thus, the current information per each nanocompound is not constant and there are some empty cells. This database is going to be dynamic, and more information is going to be included when new papers are published. This is going to be by adding more sheets in **MO-NanoDatabase.xlsx** and adding the XYZ and NanoFingerprint files of the new included nanocompounds. Note the non-metallic compound SiO2 is included in this repository, which demonstrates that it could be extended to other type of compounds.

## Ethics Statement

The authors have read and follow the ethical requirements for publication in Data in Brief. The current dataset does not involve human subjects, animal experiments, or data collected from social media platforms.

## CRediT authorship contribution statement

**Francesc Serratosa:** Conceptualization, Methodology, Software, Data curation, Writing – review & editing. **Natàlia Segura-Alabart:** Conceptualization, Methodology, Software, Data curation, Writing – review & editing.

## Data Availability

GithubMO-NanoCompound (Reference data).
